# Introducing rapid tests for malaria into the retail sector: what are the unintended consequences?

**DOI:** 10.1136/bmjgh-2016-000067

**Published:** 2017-01-11

**Authors:** Eleanor Hutchinson, Coll Hutchison, Sham Lal, Kristian Hansen, Miriam Kayendeke, Christine Nabirye, Pascal Magnussen, Siân E Clarke, Anthony Mbonye, Clare I R Chandler

**Affiliations:** 1 Department of Global Health and Development, London School of Hygiene and Tropical Medicine, London, UK; 2 Department of Social and Environmental Health Research, London School of Hygiene and Tropical Medicine, London, UK; 3 Department of Disease Control, London School of Hygiene and Tropical Medicine, London, UK; 4 Department of Global Health and Development, London School of Hygiene and Tropical Medicine, London, UK; 5 The Act 2 Project, Kampala, Uganda; 6 Infectious Disease Research Collaboration, Kampala, Uganda; 7 Department of International Health, Immunology and Microbiology, Centre for Medical Parasitology & Institute for Veterinary Disease Biology, Section for Parasitology and Aquatic Diseases, University of Copenhagen, Kobenhavn, Denmark; 8 Faculty of Infectious and Tropical Diseases, Department of Disease Control, London School of Hygiene and Tropical Medicine, London, UK; 9 Ministry of Health, Kampala, Uganda

## Abstract

The observation that many people in Africa seek care for febrile illness in the retail sector has led to a number of public health initiatives to try to improve the quality of care provided in these settings. The potential to support the introduction of rapid diagnostic tests for malaria (mRDTs) into drug shops is coming under increased scrutiny. Those in favour argue that it enables the harmonisation of policy around testing and treatment for malaria and maintains a focus on market-based solutions to healthcare. Despite the enthusiasm among many global health actors for this policy option, there is a limited understanding of the consequences of the introduction of mRDTs in the retail sector. We undertook an interpretive, mixed methods study with drug shop vendors (DSVs), their clients and local health workers to explore the uses and interpretations of mRDTs as they became part of daily practice in drug shops during a trial in Mukono District, Uganda. This paper reports the unintended consequences of their introduction. It describes how the test engendered trust in the professional competence of DSVs; was misconstrued by clients and providers as enabling a more definitive diagnosis of disease in general rather than malaria alone; that blood testing made drug shops more attractive places to seek care than they had previously been; was described as shifting treatment-seeking behaviour away from formal health centres and into drug shops; and influenced an increase in sales of medications, particularly antibiotics.

**Trial registration number:**

NCT01194557; Results.

**Key questions**

**What is already known about this topic?**

- Rapid diagnostic tests for malaria (mRDT) now form a central element of malaria care.
- The scale-up of mRDTs through retail outlets is being pursued by several governments in Eastern and Southern Africa.
- Existing evaluations focus on the intended consequences of mRDT use.

**What are the new findings?**

- This study examined the unintended consequences of the introduction of mRDTs into registered drug shops in Mukono, Uganda.
- The popularity of the tests appeared to be linked to a rise in the status of drug shop vendors.
- Drug shops with mRDTs appeared more attractive places to seek care, sales of medicines increased and treatment seeking appeared to shift out of the formal sector providers and into these shops.

**Recommendations for policy**

- Policy makers need to be aware of the unintended consequences of interventions before they decide on whether they are appropriate or not. This is especially the case in settings in which there is little regulation and oversight of practice.
- If mRDTs are introduced into drug shops in Uganda, a significant increase in surveillance and supervision is likely to be necessary in order to guard against poor quality, inequitable practices that may increase out of pocket payments among those who can least afford them.

## Introduction

Across East Africa, urban and rural citizens’ use of retail outlets to access pharmaceutical medicines to treat episodes of febrile illness is well established.^1–6^ Current framings of the global drive to control and eliminate malaria often place this observation at the core research agendas, programme financing and national decision-making processes.^7^

This interest in providing malaria medication through the retail sector has coincided with a shift in global policy from the presumptive treatment of all fevers as malaria to the targeted treatment of parasitologically confirmed malaria, a policy to be realised in large part through the introduction of a rapid diagnostic test for malaria (mRDT).^8^ For many, this has made the successful introduction of mRDTs into the retail sector imperative.^9^ ^10^ For others, the importance of introducing mRDTs has been underscored by the results of the Affordable Medicines Facility for Malaria (AMFm) project, which sought to flood private sector markets with affordable, quality assured artemisinin combination therapy (ACT), to crowd out less efficacious antimalarials.^11^ ^12^ AMFm achieved its goal of improving access through changes in the private for profit sector, but in so doing, it precipitated a substantial increase in the use of ACTs among those who did not have malaria.^13^ This raised concerns about the cost-effectiveness of the subsidy, as well as whether it would favour the development and spread of parasite resistance to artemisinin and risk the future efficacy of the drug.

Despite the fact that introducing mRDTs into private sector markets appears a pragmatic solution to the overuse of ACTs in retail outlets^9^ ^10^ and has been enthusiastically taken up by many national-level and global-level policymakers, studies on the impact of the mRDT have shown varied results and been limited in scope.^11^ ^13^ ^14^ In the retail sector in Kenya, while mRDTs were reported to have been popular among clients, they did not substantially reduce the numbers of ACTs sold to people who tested negative.^11^ In Uganda, two studies reported that it is feasible and safe to introduce mRDTs into registered drug shops. In the first study, the response to the introduction of mRDTs was heterogeneous in terms of uptake and adherence to test results.^13^ In the second study, however, mRDTs were taken up enthusiastically by registered drug shop vendors (DSVs) and ACTs were appropriately targeted at 79% of patients in the mRDT arm as opposed to 33.7% in the presumptive treatment arm.^15^

While these studies have been concerned with ascertaining whether and how mRDTs can be introduced, holistic accounts that describe the mechanisms that their successful introduction is based on and the unintended consequences of their introduction are lacking. This constitutes a considerable gap in the literature as all interventions have impacts on society that cannot be foreseen and which must be attended to in the overall evaluation of an intervention.^16^ ^17^ This is especially the case in places such as drug shops that are poorly regulated and in which poor practice is unlikely to be influenced or curtailed by government policy and surveillance.

This paper adds to the literature on drug shops and mRDTs by providing an anthropologically informed analysis of the unintended consequences of the introduction of mRDTs into the retail sector in Uganda. It explores the introduction of mRDTs into drug shops as a process of assemblage, focusing on how mRDTs were arranged, organised and interacted with other objects, people, medicines, desires, forms of regulation and everyday practice found in drug shops in Mukono District. In so doing, the paper shows that the popularity of mRDTs among both DSVs and their clients was not a straightforward process of the adoption of a technology that rationalised everyday practice. Instead, we show how the use and popularity of the test was intimately connected to local concerns about the trustworthiness of DSVs, the quality of services on offer in these shops, and the efficacy of medicines and relationship between drug shops and formal health providers.

### Theoretical background

Anthropological approaches to evaluation, with a commitment to inductive, holistic research, are well suited to analysing the consequences of programmes beyond those specified in proposals and protocols. It has been suggested by Kleinman^17^ that this type of analysis is one of the key contributions that medical anthropologists can make to global health.^18^ ^19^ His interest stems from Merton's original formulation of the unanticipated consequences of purposive social action as the unforeseen effects of actions with a motive, a choice among alternatives, a goal and a process (such large-scale government programmes, but also complex intervention trials).^16^ ^17^ Merton offers several reasons for their emergence including unforeseen shifts in context (such as ongoing economic or social changes); the immediacy of the interests of those formulating the programme; and the institutional values that shape the thoughts of programme managers or policymakers which make it hard to see beyond their particular paradigm.^16^ While this approach is useful in pointing to the complexity of introducing programmes to promote social change, it offers relatively little in terms of understanding *how* local, everyday practices shape interventions. Moreover, it offers no means of understanding how interconnections between the intended and unintended consequences of an intervention may come about and, importantly for this paper, how one may influence the other.

This paper draws on post-structuralist theories of the microprocesses of sociomaterial relations, sometimes referred to in the literature as theories of assemblage, to better understand and develop an analysis of the unintended consequences of introducing mRDTs into the retail sector. Drawing on the French philosopher Deleuze, these theories have been influential in anthropology and sociology for their ability to account for the interaction between society, science and technology and local processes of social change.^20^ ^21^ This gives ideas of assemblage an obvious relevance to the analysis of the introduction of mRDTs and also provides a pertinent frame for understanding Ugandan drug shops more generally. Like other ethnographic and social analyses of drug sellers and informal private providers,^22^ ^23^ the literature on Ugandan drug shops has shown these to be spaces that provide an awkward mix of services, combining disparate elements from different social arenas and in which those selling drugs do not necessarily adhere to a single logic (biomedical or economic, scientific or religious) but are more likely to draw on and display forms of practice that come from multiple institutional settings.^3^ ^24^ Analyses that attend to the structural position of drug sellers tend to present them as existing in an odd and often insecure position, as liminal actors occupying in an in-between social space.^3^ Post-structuralist accounts attend to the ways in which social fields are assembled rather than structured and suggest that the analyst shifts the focus. Instead of mapping the contradictions that shoot through everyday practice, findings seek to account for the ways in which heterogeneous elements of the social and material world (from medicines, to social norms, desires, people, buildings and illness) come together and cohere, influencing and impacting on one another.^25^

In terms of accounting for the intended consequences of the introduction of mRDTs into drug shops, theories of assemblage draw our attention to the ways in which programmes or projects work by establishing relationships between the mRDT and the use of malaria medicines and that they fail when they do not. Yet, as this intended relationship is established, relations between the other social and material elements within the shop emerge and it is these processes (sometimes quite self-consciously arranged and organised by DSVs and their clients) that create the unintended consequences of the intervention.

A focus on the unintended consequences through the lens of assemblage theories therefore makes the connections (or sociomaterial relations) between already existing elements of the drug shop and those that are put in place through the actions of those implementing a project or programme the key sites for analysis. Through a focus on everyday practice, this paper analyses the processes through which mRDTs became part of the assemblages that constitute the drug shops in Mukono. Combining qualitative and quantitative data, it explores three sets of connections that were key to shaping the uses of the mRDT in the drug shops: local markets, regulation, diagnosis and trust; blood tests, disease and the generation of trust between DSV and their clients; mRDTs, pharmaceutical markets and the desire for effective medicine.

### Study setting

Between October 2010 and December 2011, a cluster-randomised trial was carried out in 59 registered drug shops grouped into 20 geographical clusters in a periurban area in Mukono, Uganda. Staff from 65 drug shops had been invited to join the trial, though 6 shops failed to attend the training and so were excluded. Following invitation and consent to join the project, clusters of drug shops were randomised to receive training in one of two methods to diagnose malaria: 10 clusters were trained to use clinical signs and symptoms (the presumptive arm), and 10 clusters were trained to use mRDTs (the mRDT arm). All DSVs attended a 3-day training in taking patient history, recognising malaria, prescribing ACTs and rectal artesunate, when and how to refer patients into the health service. In addition, the DSVs in the intervention arm received an additional day’s training on when and how to use an mRDT. DSVs were also trained in record keeping and preparing blood slides for the reference microscopy conducted on each client, which were used by the trial to evaluate whether the treatment decisions were appropriate or not. All DSVs were given ACTs for free (artemether-lumefantrine) to sell to patients at an agreed low retail price (child: 1000 Uganda shilling, adult: 3000 Uganda shilling, equivalent to US$0.40 and US$1.20, respectively), disposable gloves, cotton wool, lancets, blood slides, safety boxes for sharps disposal, triplicate carbon copy patient registers for record keeping for the project, and to provide a referral form should the client be considered too ill to be treated in the drug shop. All DSVs in the mRDT arm were additionally provided with a continuous supply of mRDTs for free, and were asked to sell these to patients at a fixed price (500 Uganda shilling, equivalent to US$0.20). The DSVs in the mRDT intervention arm were provided with signs to place outside their shop advertising the availability of mRDTs. In both arms, community sensitisation through Village Health Teams informed local residents about mRDTs underlining the fact that not all fevers are malaria; that it was beneficial to test for malaria before providing treatment and that mRDTs were available locally from trained DSVs and public health facilities. A detailed description of the various elements of the intervention is provided in the main trial paper.^15^

## Methods

Towards the end of the trial, between November 2011 and February 2012, research was undertaken to provide an anthropologically informed, holistic account of the intervention by exploring the processes and everyday practices involved with the introduction and incorporation of mRDTs into drug shops and their affect on the referral of clients from drug shops to other health facilities. The research was particularly concerned with the relationships between the different elements of the trial, and the ways in which they impacted on the local context of health seeking, the provision of healthcare and the perception and experiences of conducting an mRDT among providers and care seekers. The methods used reflect the COREQ recommendations for qualitative research^26^ and the relationships between the researchers and the participants have been described in detail elsewhere.^27^

### Focus group discussions

Focus group discussions (FGDs) were conducted with three subgroups: drug shop clients, DSVs and health workers at local public facilities and a private not for profit hospital. In all, 21 FGDs were conducted (see table 1) and each FGD had at least 5 and no more than 13 participants.

**Table 1:** Focus group discussions: numbers, participants, recruitment, stratification and exclusion criteria

	DSV	Health worker	Drug shop client
Number of FGDs in the RDT arm	4	5	4
Number of FGDs in the presumptive arm	3	3	2
Total FGDs	7	8	6
Total FGD participants	54	71	54
Recruitment method	From project records	From visits to the health facilities	From project records
Stratification	By arm and frequency of referral	By arm and level of health facility	By referral for carers (for children) or adult seeking care for febrile illness; or those who had visited a shop for care for febrile illness
Exclusion criteria	Those who had worked in the area for <6 months	Those who had worked in the area for <6 months	Those who had not visited a drug shop in the past 6 months, children under the age of 18

DSV, drug shop vendor; FGD, focus group discussion; RDT, rapid diagnostic test.

The participants were given a description of the project when they were invited to participate in the FGD. On the day of the FGD, the project was described again and the participants were asked to sign a sheet to consent in the research. They were advised that at any point they could leave the FGD and that there was no requirement for them to stay. Focus groups were conducted in Luganda and/or English, and in all FGDs notes were taken and the discussion recorded digitally.

The FGDs were conducted in a Sunday school building except for two that were conducted at health facilities. Each focus group was attended by three social scientists acting as facilitator, note taker or organiser of the session. The topics discussed were local treatment-seeking behaviour, experiences at drug shops, experiences with rapid diagnostic tests and experiences with referral. The latter was a particular interest in the study and this focus on referral from drug shops into public sector health facilities was the reason behind the inclusion of health workers from local health centres and hospitals in the research. An emphasis was placed on describing and understanding actual experience rather than eliciting generalised or ideal statements. The facilitators also allowed conversations to develop between participants when important but unanticipated themes arose. Participants received money to cover the cost of their travel and refreshments.

Recordings were transcribed, translated in English and imported into Nvivo (QSR International, Cambridge, Massachusetts, USA), a qualitative data analysis software package for the aggregation of data by codes.

Coding and analysing qualitative data is an interpretive endeavour and each FGD was coded by one social scientist who had collected the data with close support and supervision from the anthropologist (EH). A coding scheme for each group was drawn up through a combination of themes emerging from the transcript coupled with predefined areas of interest identified from the main intervention (mostly the interaction between different components of the trial) and from anthropological theory on informal health providers. The coding scheme was adapted as necessary to reflect new themes emerging and the anthropologist provided input at the conceptual level of analysis. The main findings were discussed with the principal investigator of the main trial, the anthropologist and the project's social scientists during a series of face-to-face meetings that took place everyday over the course of a week.

### Follow-up questionnaires

DSVs in all clusters recorded the names and contact details of clients who visited their shops for a fever or history of fever. Interviewers then visited DSVs according to a randomised schedule to identify recent clients and carried out questionnaire interviews (n=646) at the client's home after 4 days, and again between 10 and 14 days after visiting a drug shop. The questionnaires were conducted to elicit reports on the economic costs associated with treatment of the recent febrile episode at a drug shop, whether they had been offered and had accepted to be tested with an mRDT, their reported results; the type and quantities of drugs purchased and any subsequent costs over the next 10–14 days; and exploring perceptions of mRDTs, their knowledge and understanding of the tests, treatment-seeking behaviour and referrals. For clients who were under 18, questionnaires were conducted with the adult who attended the drug shop with the child; clients under 18 who attended the DSV alone were excluded from the study. Clients who did participate or consent to both questionnaires or did not meet the overall study inclusion criteria were excluded from the follow-up analysis. The analysis of open questions was coded by CH using Nvivo (QSR International, Cambridge, Massachusetts, USA) and, where appropriate, imported into Stata V.13 (StataCorp LP) and converted into ordinal or categorical variables for descriptive quantitative analysis in conjunction with quantitative responses.

Ethical clearance was granted by the London School of Hygiene and Tropical Medicine and the Uganda National Council for Science and Technology.

## Results

### Context: diagnostic practice and the generation of trust

According to government policy, registered drug shops are places in which over-the-counter medication may be bought and sold. Legally, drug shops are distinct from private clinics and expected to provide a limited range of products for sale, which excludes prescription medication such as antibiotics. In Mukono, however, categories of drug sellers were fluid and clients were unable to categorise private sector providers effectively during FGDs. The fluidity of this categorisation was also reflected in the heterogeneous nature of the drug shops in the trial. Most DSVs reported that they provided diagnostic advice and sales of medicine only but at least 2 out of the 59 study shops also offered in-patient services and provided intravenous medication. At some, the registered owner (who by law had to have some formal biomedical training) would be in the shop for most of its opening hours, providing relatively constant access to a health provider.

As has been reported elsewhere among clients in Uganda, the care seekers attending shops in Mukono appreciated the good social relations often found in these spaces^3^ but maintained a generalised suspicion of DSVs’ motives and skills.^27^ DSVs could be respectful and kind; attentive to the psychological, social and spiritual needs of their clients; understanding of and responsive to their economic constraints (providing of credit and selling cheaper or incomplete doses) and keen to use their skill and expertise. As one woman who had visited a drug shop in the mRDT arm of the trial with her child explained, these social relations often revolved around a desire for powerful pharmaceutical drugs to manage illness in them and in their children.

We had five thousand shillings (2 US$) in our pocket. But because the sickness was severe, she wrote us a bill of eighteen thousand shillings (7 US$). Yet, she had given us a plenty of drugs in a polythene bag. Then I told her, “I have come with five thousand [shillings],” and she said, “Aha, there is no problem. You can even come back tomorrow”, and she didn't even know us…[later in the discussion]…This health worker gives you drugs and you also look at it and say, “as I have never seen such a quantity of drugs!” and you simply say that. Oh, but the drugs are worth the money you pay!

FGD with mothers in the mRDT arm of the trial

Attending a drug shop was also a risky act. Businesses were infrequently visited by government regulators. In all three groups of participants in the FGDs, the following narratives echoed: drug shops are often run by those who claimed expertise but had none, who knowingly sold stolen, dangerous, expired or counterfeit drugs and were only interested in making as large a profit as possible. Accounts of particular instances of receiving poor quality care at drug shops and its consequences for children's health were commonplace. As previously described by Birungi,^28^ injections were a popular form of medicine, a sign of good care but raised particular concerns about the infection and illness that could be caused when unskilled DSVs administered injections to small children. Moreover, in the context of ongoing concerns about how to pay for treatment, clients expressed concern that they could be overcharged for medication by exploitative DSVs.

Where some FGD participants considered it to be almost impossible to distinguish between skilled and unskilled, trustworthy and untrustworthy DSVs, others described how they acted to protect themselves against the risks associated with attending a drug shop. Previous treatment success, recommendations from neighbours and the DSV's diagnostic assessments played a central role in identifying an effective, good quality and caring provider. Below, a group of women described how they evaluated a DSV's skill through the questions that s/he asked about the child's symptoms.

P6: When you look there, when you are just passing by, or maybe if you have had your child fall sick, some people base [their assessment of a drug shop] on the way the drug shop looks and then see whether they [the DSVs] are skilled, drugs are in plenty. But, when you pass there, [you may see that] the drugs that are there are few. Now you can despise the place yet in the real, actual sense she has the idea.

SS1: What do you mean by that thing of ‘idea’?

P2: What she wants to say is that she knows what she does.

SS2: How can you come to know that someone is knowledgeable?

P6: For me what I can explain, you can take a child and you tell her, “Health worker, I feel the child has high temperature.” You have not yet told her that it seems the child is sick with malaria or what but for her she starts asking you that…that is to say she knows the conditions of children. Most of the time she asks you, “Has he done this, do you feel his head is a bit hot (some high temperature), how was he at night, did he at any time have diarrhoea?” Okay things like that. That is to say, you have not told her but she says correct things and you tell her, “yes, health worker that is how it was”, before she measures his temperature to see the temperature…and she asks you about the condition of the child, or how he was, whether it is malaria or it is another thing.

FGD with mothers in the non-RDT arm

The diagnostic process underway in these drug shops that existed prior to or without the introduction of mRDTs had a productive role that went beyond the imperative of identifying disease in order to provide treatment. In this heterogeneous and poorly regulated market, it was an important demonstration of medical knowledge, and acted on the relationship between the DSV and the client, engendering trust in a space in which questions around motivation of the vendor and the type of service that they provided were under constant scrutiny.

### mRDTs’ impact on decision-making about where to seek care

According to the FGDs, mRDTs had not been available in the drug shops in Mukono prior to the intervention but when they were introduced, they were popular among DSVs and clients. Characteristics of the clients interviewed on day 4 after drug shop consultation are presented in table 2. Following refusal of consent (12) and loss to follow-up (130), clients or carers comprised 251 who had visited a drug shop in the presumptive diagnosis arm and 253 in the mRDT arm of the trial. In both arms of the trial, half of those seeking care and medication from a drug shop were children under the age of 5, at 49.4% in the presumptive arm and 51.8% in the mRDT arm. Of the 253 clients presenting with symptoms of a febrile illness in the mRDT arm, 97.6% reported that they had purchased an mRDT. There were occasional reports of mRDTs also being used in the presumptive arm (7/251 cases), findings that correspond to those from the FGDs. This suggests that certain DSVs were making the choice to purchase and offer these tests for sale (see table 2).

**Table 2:** Characteristics of drug shop clients followed up and interviewed at home 4 days after the drug shop visit

	Clients who visited drug shops in the presumptive arm N=251		Clients who visited drug shops in the RDT arm N=253	
	n	Per cent	n	Per cent
Age of client (years)				
0–1	28	11.2	42	16.6
1–5	96	38.2	89	35.2
5–18	75	29.9	54	21.3
18–45	40	15.9	49	19.4
45+	12	4.8	19	7.5
Sex of participant				
Female	137	54.6	122	48.2
Male	114	45.4	131	51.8
Role of respondent				
Client (18+)	45	17.9	59	23.3
Mother	157	62.5	141	55.6
Head of household	19	7.6	22	8.7
Other guardian/carer*	30	12.0	31	12.3
Reported symptom(s)				
Diarrhoea	23	9.2	23	9.2
Cough or influenza	93	37.0	90	35.7
Fever	130	51.8	115	45.6
Headache	57	22.7	50	19.8
Vomiting	19	7.6	34	23.5
Malaria	26	10.4	21	8.4
Went elsewhere before this DSV				
Yes	107	42.6	114	45.1
No	140	55.8	128	50.6
Did not report anything	4	1.6	11	4.3
Purchased an mRDT				
Yes	7	2.8	247	97.6
No	244	97.2	6	2.42

*Where the adult patient or carer of the sick child was not available for interview, and the household head identified themselves as sufficiently knowledgeable about the illness episode to be able to answer questions about it.

DSV, drug shop vendor; mRDT, rapid diagnostic test for malaria; RDT, rapid diagnostic test.

Almost three-quarters of these care seekers in the mRDT arm of the trial (72% of the 253 respondents) reported in the questionnaires that they knew about the availability of mRDTs prior to the recent visit to the shop. Of those, the majority (73% of the 172 respondents) considered that this had an impact on their decision-making and why they had chosen to seek care in a particular drug shop. During the FGDs, health workers also noted changes in treatment-seeking patterns.

P4: It [the introduction of mRDTs] has not affected us badly because it has helped to reduce the number of patients [that we see] and they [the DSVs] also get some money…[laughter]…

P2: In my opinion, if a person has a sick child and they go to the drug shop and they take off some blood in order to really confirm that she has malaria they get to know the truth. They [the client] are happy because instead of going to the hospital they are being treated nearer their home.

*FGD with health workers from HC II in the intervention arm site*

P5: We now get fewer cases of malaria at our hospital. You know what that means for our income, which is a bit negative for us but for the community it's okay because they have the services nearer.

FGD with health workers in HC IV and the not-for-profit hospital

As mRDTs were introduced into drug shops, the process of finding out about disease maintained its ability to engender trust between the vendor and the client and the benefits of mRDTs were interpreted through this lens. The first quotation below links this to a process of being able to see and then act on a disease, while the second connected the care available at drug shops to the larger health facilities.

Okay for me the different thing that I have seen, we have been suffering and what made us like the hospital are the blood tests. Yet, in these drug shops, they would simply inject you without testing your blood to see what type of fever it is. But for me the different thing that I have seen now, [is that] they [DSVs] test blood. Now we have come to completely dislike the public sector hospital because that [blood testing] is what has been mostly taking us there.

*FGD with mothers in the mRDT arm of the trial*

P1: The majority of us used to go to those big health facilities but now we go there to drug shops and clinics and they give you treatment.

P5: I think it [testing] has been good for them [the drug shops vendors] because the majority of people have trust in them. Previously, they would despise those clinics.

Facilitator: Previously you never used to put trust in them. Why were you going to other health facilities in the past?

P4: Blood, they never used to test blood.

Many Ps: Hmmm, blood

FGD with mothers in the mRDT arm of the trial

Refracted through this new logic of testing before treating illness, previous diagnostic practices in the drug shop were conceptually reconfigured. The practice of describing and suggesting symptoms so that they could be matched to available medicines was often recast as guess work and was contrasted with the definitive knowledge that the mRDT provided.

I think work has been simplified because those days, before this project started, we would just try to guess what's going on, how to treat the patient. But after testing, I think we know exactly what we are doing and even the patients themselves realize it and that's why they come. But of course, those days they [patients] would have that negative…what? [attitude]…but now they know if you go there, they test you and they give you…what?…[They] will cure you. Of course that confidence has increased in them. Ya, they are confident that they going to get what? [That they are going to get] okay.

FGD with DSVs in the mRDT arm of the trial

The claims by DSVs and clients of the benefits of using mRDTs were significantly broader than those stated in the project protocol. A few clients reported that the mRDTs in the shops they visited diagnosed malaria, typhoid and syphilis (eg, “what came out of the test was that my child had malaria and syphilis,” *FGD with mothers in the mRDT arm of the trial*). Mostly, however, the capacity of the tests was not specified but was described by DSVs and their clients as creating a general change in practice that enabled DSVs to know the causes of and necessary treatment for ‘illness’ rather than just identifying those patients with/without malaria.

Ever since we started this project, it has made us treat the patients well because before we have been treating a person simply. A person comes, “I am suffering from malaria.” You would touch on him, measure the temperature and then give him drugs. But now you treat something you know and he also leaves understanding that really, they have treated me because you tested his blood.

FGD with DSVs in the mRDT arm of the trial

From the questionnaires in the mRDT arm of the trial, just under half who had received an mRDT reported that they had not seen the results of the test (see table 3). Moreover, when they reported on their interpretation of the role in the care-seeking process, 47% of the 253 care seekers reported that the use of a test either enabled the DSV ‘to know’ what was causing their illness or fever, or ‘to treat what they know’.

**Table 3:** Client reports of perceptions and experiences with mRDT

	Presumptive arm N=7		RDT arm N=247	
	n	Per cent	n	Per cent
Report being shown the mRDT result				
Yes	0	0	129	52.2
No	7	100	116	47.0
Did not report anything	0	0	2	0.8
Reported the mRDT result				
Positive	4	57.1	142	57.5
Negative	0	0	80	32.4
Did not know	3	42.9	22	8.9
Did not report anything	0	0	3	1.2
Reported the role of the mRDT as providing				
Definitive diagnosis of malaria	2	40	88	35.6
Knowledge on illness of patient	5	60	115	46.6
Did not report anything	0	0	44	17.8

mRDT, rapid diagnostic test for malaria; RDT, rapid diagnostic test.

Reports of the use of the mRDT to enable a definitive diagnosis (ie, of any illness) emerged in focus groups with DSVs and clients. In the quotation below, a mother who attended a drug shop in the mRDT arm underscores how the mRDT was imagined as enabling decisive knowledge, the effective targeting of treatment and that this would provide better value for money than in a system where malaria was treated presumptively.

That five hundred shillings [for the mRDT], and she treats what she has known. Because some time back someone could come and she (DSV) would say, “it seems that he has malaria,” and she would prescribe different drugs for you and she would tell you that you had to pay this and this amount of money. But now, she takes blood from you and she gets to know that she has tested your blood and you have malaria and the drugs she gives you are for malaria.

FGD with mothers in the mRDT arm of the trial

The DSV and the client quoted above also provide details of what makes up a good visit to a drug shop, that the client has been tested and purchased effective medication. The final section of the results considers the ways in which mRDTs acted on clients’ desires for effective medicines and reshaped local pharmaceutical markets in Mukono.

### mRDTs increase medicine sales in drug shops

Like many of those working on the introduction of mRDTs in other sectors of health systems in Africa, the researchers involved in the mRDT trial in Mukono were concerned with the ways in which DSVs would manage negative test results. A negative mRDT could mean that drug sellers would not make a sale, reducing their income, and disincentivise testing among DSVs. We have previously shown how RDT use can also affect a DSV's reputation.^27^ Negative results carry a risk of revealing diagnostic uncertainty which could be interpreted as a lack of knowledge and skill in managing a patient's illness. During the FGDs, some clients did express concern about the ability of the test to always provide an accurate result. During the FGDs with the DSVs, however, concerns about negative test results were relatively rare. A close look at the interpretation of the capacity of the mRDT, its perceived ability to create definitive knowledge around disease and the way in which its introduction set a new standard for care based on testing blood helps to make sense of this.

When DSVs described anxiety around the management of a negative mRDT result, this was most often linked to encounters with clients who were convinced that they had malaria and wanted to purchase *an ACT*, but what also appears to have been critical in managing a negative result was the action taken by the DSV following the generation of the result. Referring a client who was not considered to be dangerously sick, especially if the DSV did not provide any medication first, was considered to be economically perilous and also hazardous to their reputation and so was infrequent.^27^ Instead, DSVs could act on a negative test result by providing what was understood by the client to be effective treatment. Below, two different mothers from different focus groups explained a positive outcome of their trip to the drug shop using mRDTs with their children. In the first description, the child is diagnosed with malaria, and in the second the child tests negative to malaria but both received an injection and other medication.

After testing the child's blood, she gave him an injection and ended there. The malaria was very severe and she gave him an injection. She even gave me tablets that were yellow and we went back home. She even told me that I have to give to the child plenty to drink.

*FGD with Mothers in the mRDT arm of the trial*

For me, I took a child there [to the drug shop] who had diarrhoea and had fever. They tested him but there was no malaria. They gave him drugs and syrup and they also gave him four injections. They gave him four injections and he got well.

FGD with Mothers in the mRDT arm of the trial

The imperative to receive medicines as an outcome of drug shop encounters in both arms of the trial was underscored by data from the questionnaires with care seekers, where 100% of those interviewed during follow-up reported having purchased some form of medication (table 4). Unlike in the FGDs, where medicines were often identified by their colour, whether they were in tablet form, syrup, injection or crushed into a drink, for the questionnaires clients were asked to specify wherever possible the type of medicine that they had purchased. In concordance with the overall findings from the trial,^15^ reported sales of ACTs were substantially lower in the mRDT arm than in the presumptive arm, though not entirely ruling out the sale of malaria medicines to patients with a negative test. In the mRDT arm of the trial, for the clients who reported having a positive mRDT result, the majority said that they had purchased an ACT (94%) either with (17.9%) or without (76.4%) an antibiotic. A similar percentage to those being treated for febrile illness in the presumptive arm purchased an antibiotic (18%; see table 4). As one of the FGD respondents explained, past experiences of giving malaria medication to her son during an illness episode continued to influence the clients’ preference and demands for particular medicines.

**Table 4:** Treatments purchased in registered drug shops by method of diagnosis and mRDT test result

Type of drug treatment	mRDT arm			
	mRDT negative N=101 (%)	mRDT positive N=123 (%)	RDT total (%)	Presumptive arm N=246 (%)
No drugs purchased	0 (0.0)	0 (0.0)	0 (0.0)	0 (0.0)
Antipyretic only	22 (21.8)	4 (3.3)	26 (11.6)	0 (0.0)
Other drugs only (eg, cough syrups, deworming)	13 (12.9)	1 (0.8)	14 (6.3)	0 (0.0)
Antibiotic without any antimalarial	29 (28.7)	0 (0.0)	29 (12.9)	1 (0.4)
Other non-ACT antimalarial treatment without antibiotic	4 (4.0)	2 (1.6)	6 (2.7)	0 (0.0)
Other non-ACT antimalarial treatment with antibiotic	3 (3.0)	0 (0.0)	3 (1.3)	1 (0.4)
ACT without antibiotic	24 (23.8)	94 (76.4)	118 (52.7)	210 (85.4)
ACT with antibiotic	6 (5.9)	22 (17.9)	28 (12.5)	34 (13.8)
Median cost (UGX) (minimum–maximum)	5000 (200–33 000)	4050 (300–32 000)	5000 (200–33 000)	3300 (800–45 000)
Median cost US$ (minimum–maximum)	2 (0.08–13.20)	1.62 (0.12–12.80)	2 (0.08–13.20)	1.32 (0.32–18.00)

ACT, artemisinin combination therapy; mRDT, rapid diagnostic test for malaria; RDT, rapid diagnostic test.

Now, when you get to the health worker he tests and tells you there is no malaria and as a result you tell him, “Health worker, the other time you gave me these tablets and these tablets. So, still just give me maybe Camoquine or Chloroquine and I go and give [them to] him and I see if he will get better.”

FGD with mothers in the mRDT arm of the trial

While the results of the FGDs suggest that there was a good deal of polypharmacy among those testing both positive and negative for malaria, the questionnaires suggested that the range of medicines sold to clients with a negative test result was greater than either those with a positive result or those who had not been tested at all. It appears that more mRDT-negative clients purchased an antibiotic compared with mRDT-positive or untested patients—37.6–18% and 15%, respectively. Overall, the median amount of money spent by clients in drug shops using mRDTs was higher than those in the presumptive arm and the difference was marginally greater among clients in the mRDT arm who received a negative test result.

## Discussion

This paper has provided an analysis of the unintended consequences of the introduction of mRDTs into drug shops in Mukono district, Uganda. The study took place alongside a cluster-randomised control trial with the intention of providing an analysis of the broader impacts of introducing rapid diagnostic tests in drug shops. For the FGDs, our sampling strategy ensured that we included a range of participants providing and seeking care within Mukono and the semistructured interviews were based on a random sample. In both cases, however, the study was limited by its use of data that relied on participants’ own accounts of their practices and experiences and it is possible that both the FGD participants and those responding to the interview questions shaped their responses in a particular way that did not always reflect actual practice. The approach could have been enhanced by incorporating participant observation, thus enabling researchers to observe the dynamic interactions between test results, medicines, formulations of expertise and good quality care.

To conduct the analysis, we analysed qualitative and quantitative data, using the lens of assemblage theories to prompt a focus on the ways in which mRDTs were arranged and organised *in relation to* other objects, people, medicines, desires, forms of regulation and everyday practice found in these spaces. In so doing, we suggest that the success of the intervention and its intended effect of facilitating high rates of uptake of the test and adherence to mRDT results was most likely connected to the development of trust between the DSVs and their clients, increased sales of unauthorised medicines and the attraction of clients to drug shops during illness episodes which would have previously resulted in a trip to the public and private not for profit formal sector.

Our findings that the introduction of mRDTs appeared to make drug shops more attractive places to seek care resonate with other observations from the main trial. In comparison with both the exit interviews conducted before the trial began^29^ and results from the presumptive arm in the main trial, a significantly higher proportion of clients seen by DSVs in the mRDT arm were parasite positive by microscopy, suggesting that there may have been a differential change in treatment-seeking behaviour following the introduction of mRDTs (Mbonye, personal communication). These are different from research findings in Kenya in which the introduction of subsidised ACTs in drug shops, rather than the introduction of subsidised mRDTs, was considered to impact on decision-making about where to receive care.^11^ These results, however, came from a study in which the results of mRDTs overall were reported to have had little impact on decision-making about treatment and the authors suggested that this lack of effect on treatment seeking was likely to reflect their limited impact on treatment decisions more generally.^11^

Our findings challenge widespread concerns that adherence to negative mRDT results would be undermined in the retail sector because of the importance of making a sale in these places.^9^ First, recorded adherence to positive and negative mRDT results in relation to the prescription of ACTs was high in the main trial findings.^15^ Second, questionnaires with clients found that none had left the shop empty-handed and that costs to clients were higher in the mRDT arm of the trial. Reported sales of antibiotics were substantially more common in patients who tested negative, with a slight increase also in non-ACT antimalarials, suggesting that the ability to sell other medications (including the illegal sale of antibiotics and injections) was a factor in facilitating adherence to negative test results.

Finally, the perception that mRDTs can identify other causes of illness and not just malaria has also been noted in other settings. In a qualitative study of patients receiving care in public sector facilities in Ghana, the authors were concerned that once patients learnt of its limitations, this would lead to disappointment with the test.^30^ Work on the acceptability of introducing mRDTs into drug retail outlets in Kenya also suggests that the capacity of mRDTs may be misinterpreted in these settings—drug sellers there reported that their introduction would be able to remove the ‘guesswork’ from diagnostic practice (with no reference to malaria per se), enabling drug sellers to target drugs more effectively.^10^ Whether the mRDT will appear as attractive to DSVs or their clients if its limitations were better understood remains an important area for further investigation. Further research will also be needed to ascertain whether the trust in the test as a decisive tool through which disease can be known will change and diminish over time and the effects that it will have on the attraction of the test to DSVs and their clients.

## Conclusion

Interest in the potential of markets and the retail sector in particular to deliver healthcare in low-income settings is evident across many debates within global health.^31^ For those concerned with improving access to quality assured, targeted treatment for malaria, investing in public and donor funds in the retail sector through subsidies for first-line antimalarial medication and mRDTs appears a logical step.^9^ ^10^ ^32^ ^33^ While the retail sector is constituted differently in different countries, in many low-income countries the lack of regulatory oversight of drug shops makes the need for holistic accounts of interventions in this sector imperative before policy change is put into effect. In this paper, we constructed such an analysis by drawing on Merton's idea of the unintended consequences of purposive action and recent theories of assemblage. We have shown how the mRDT interacted with the desires of care seekers for trustworthy providers and targeted pharmaceutical cure for their illness; and the lack of regulation of medicine sales makes the mRDT appear more powerful than it is and drug shops more attractive places to seek care. Once these dynamics are taken into account, national policymakers may consider that introducing mRDTs will have to go alongside significant concomitant improvements in the regulation of drug shops. These could possibly be achieved through accreditation schemes^34^ ^35^ and/or through the workings of national health insurance programmes that may come into place in some low-income settings over the next decade.
